# Ontario Veterinary Medical College Society

**Published:** 1898-02

**Authors:** C. W. Fisher

**Affiliations:** Secretary; Toronto, Ontario


### ONTARIO VETERINARY MEDICAL COLLEGE SOCIETY.

THE following is a list of the papers read during the past month at the meetings of the Society. Many of the essays have shown thorough work on the subjects discussed, and the communications have brought forward several rare cases for discussion, thus giving the students the advantage of hearing from a very wide field of practice :

*Essays.* J. C. Palmer, Osteoporosis; J. W. Rutledge, Diagnosis of Disease; G. T. Elliott, Torsion of Uterus; J. P. Stranghan, Cryptorchids; J. E. Sexton, Fistulous Withers; A. J. Cromwell, Dehorning; A. Jordan, Quittor; J. L. Devereau, Polyuria; A. Sorenson, Dermititis Gangrenosa; R. B. Coults, Future of Veterinary Medicine; G. T. Irons, Administration of Medicine; W. J. Ackerman, Choking; F. J. Neiman, Congestion of Lungs; Wm. Cunningham, Loco-Poisoning; D. H. McKay, Sunstroke; J. D. Bell, Tuberculosis; E. B. Shaw, Texas Fever; L. T. Dunn, Fistula; J. T. Shannon, Glanders; D. McKenzie, Symptomatic Anthrax; W. M. Wilson, The Use of Leisure; H. R. Clark, Castration; E. C. Etwes, Texas Fever.

*Communications.* W. J. Ackerman, Azoturia; W. M. Wilson, Impaction of Rumen; P. Le C. Gauntt, Purpura Hemorrhagica; J. A. McDonald, Dorsal Injury; J. G. Cruikshank, Amputation of Penis; H. P. Reed, Azoturia; E. H. Lawley, Acute Indigestion; J. E. Sexton, Fracture of Os Suffraginia; A. J. Cromwell, Meningitis; W. H. Cory, Actinomycosa; J. L. Short, Parturient Paralysis; R. B. Coults, Injury in Region of Forearm; L. Bailey, Azoturia; W. G. Huyett, Pica in the Ox; J. T. Shannon, Parturient Apoplexy; W. Cunningham, Spasmodic Colic; G. K. Cranston, Purpura Hemorrhagica; A. D. McLachlan, Rumenotomy; A. C. Walker, Cæsarean Section; D. Allen, Hæmoglobinuria; G. T. Irons, Caries of Incisors.

C. W. Fisher,

Secretary.

Toronto, Ontario, January 13, 1898.

